# Association of simple snoring and myogenous temporomandibular disorders based on polysomnographic examination

**DOI:** 10.22514/jofph.2026.017

**Published:** 2026-03-12

**Authors:** Helena Martynowicz, Marta Bort, Dorian Nowacki, Weronika Frosztega, Jakub Przegralek, Jaroslaw Nowak, Katarzyna Madziarska, Mieszko Wieckiewicz

**Affiliations:** ^1^Clinical Department of Diabetology, Hypertension and Internal Diseases, Institute of Internal Diseases, Faculty of Medicine, Wroclaw Medical University, 50-556 Wroclaw, Poland; ^2^Department of Experimental Dentistry, Wroclaw Medical University, 50-425 Wroclaw, Poland; ^3^Department of Human Nutrition, Wroclaw University of Environmental and Life Sciences, 51-630 Wroclaw, Poland; ^4^Department of Neurology, Wroclaw Medical University, 50-556 Wroclaw, Poland

**Keywords:** Snoring, Muscle pain, Myofascial pain, TMD, Polysomnography

## Abstract

**Background**: This study aims to evaluate the association between objectively measured snoring characteristics and masticatory muscle pain in patients with myogenous temporomandibular disorders (TMD), while excluding patients with obstructive sleep apnea. **Methods**: This prospective study included 184 patients (mean age: 33.92 ± 10.05 years; 71.2% female) who underwent overnight polysomnography (PSG) and standardized TMD assessments. Snoring was quantified using acoustic recordings and parameters derived from PSG. Muscle pain intensity was assessed in the bilateral masseter and temporalis muscles. Correlation analyses and group comparisons were performed to examine the relationships between snoring characteristics (*e.g.*, snore index, train frequency, and audio volume) and pain outcomes. **Results**: No significant associations were found between primary snoring parameters and pain intensity. However, several snoring metrics,particularly those measured during specific body positions and sleep stages, especially during nonsupine rapid eye movement (REM) sleep—showed significant negative correlations with pain, mainly in the left masseter and temporalis muscles. Notably, higher snore intensity was associated with lower muscle pain, suggesting a potential modulatory effect. These relationships were lateralized and dependent on body position. Multivariate analysis did not identify independent predictors of pain. Although no direct link was observed between overall snoring and masticatory muscle pain, certain snoring patterns,particularly during nonsupine REM sleep—were inversely related to pain intensity. **Conclusions**: These findings suggest a possible protective or modulatory role of snoring in TMD-related muscle pain and highlight the complex influences of sleep stage, body position, and laterality. **Clinical Trial Registration**: Information on clinical trial registration can be found at www.ClinicalTrials.gov (identifiers: NCT03083405, NCT04214561).

## 1. Introduction

Temporomandibular disorders (TMD) are defined as a group of diseases and disorders characterized by alterations in the structure, function, or physiology of the masticatory system and can be associated with other systemic and comorbid medical conditions [1, 2]. They often present as persistent orofacial and masticatory muscle pain, jaw dysfunction, and reduced quality of life [3, 4]. Clinically, TMD represent a significant cause of non-dental orofacial pain and functional limitations, affecting approximately 29% of the general European population [5], with studies reporting a prevalence as high as 55.9% in the urban Polish population [6]. Masticatory muscle pain—the most prevalent TMD subtype, known as myogenous TMD—is particularly impactful due to its chronic nature and its association with central sensitization, somatization, anxiety, and depressive symptoms [7, 8].

Emerging evidence indicates a bidirectional relationship between myogenous TMD and sleep disturbances, including insomnia and sleep-disordered breathing (SDB) [9, 10, 11, 12, 13]. Even in the absence of obstructive sleep apnea (OSA), patients with myogenous TMD frequently report fragmented sleep, increased nocturnal arousals, and non-restorative sleep [14, 15, 16, 17, 18]. Chronic poor sleep can exacerbate musculoskeletal pain by impairing pain modulation pathways, increasing sympathetic activity, and promoting systemic inflammation [19].

Simple snoring (SS)—the audible vibration of the upper airway during sleep—is a common symptom of airway resistance and OSA [20]. It affects a substantial proportion of adults, with prevalence estimates ranging from 20% to 40%, and its prevalence increases with age, body mass index, and male sex [21, 22, 23]. Although often considered benign, habitual snoring is increasingly recognized as an indicator of airway resistance and a potential precursor to OSA [24]. While snoring itself is not classified as a disorder, it serves as an important marker of sleep-disordered breathing and has clinical implications for the craniofacial and masticatory systems, including xerostomia (dry mouth), increased susceptibility to dental caries, and temporomandibular joint abnormalities [25].

Snoring can also be associated with sleep bruxism (SB)—a sleep behavior characterized by rhythmic or nonrhythmic masticatory muscle activity [26]. Although the relationship between sleep-related conditions and temporomandibular disorders is complex and remains debated, recent systematic reviews offer further clarification. Specifically, while evidence regarding the association between sleep bruxism and TMD remains inconsistent and often conflicting, more robust and positive relationships have been reported for obstructive sleep apnea and overall sleep quality. These conclusions are based largely on studies in which TMDs were assessed using standardized Research Diagnostic Criteria for Temporomandibular Disorders (RDC/TMD) criteria, and in which the majority of available research has been rated as fair to good in methodological quality [27]. Nonetheless, the interaction between sleep-related factors, muscle activity, and pain remains an important area of investigation.

Despite growing evidence of a bidirectional relationship between sleep disturbances and TMDs, key gaps remain in understanding the specific role of simple snoring (SS)—a common yet often overlooked sleep-related phenomenon. Most current research has focused on broader categories of sleep-disordered breathing, such as OSA, and their associations with chronic musculoskeletal pain. However, studies specifically examining the impact of simple snoring—particularly in the absence of OSA on masticatory muscle pain are lacking. Although snoring is frequently viewed as harmless, emerging findings suggest that it may contribute to increased airway resistance, sleep disruption, and subtle neurophysiological changes that could influence craniofacial muscle function [28, 29]. To date, no studies have thoroughly investigated whether simple snoring, as a distinct clinical sign, is independently associated with more severe muscle-related TMD symptoms, particularly masticatory muscle pain. Additionally, the relationship between simple snoring and sleep bruxism—as well as their combined impact on the masticatory system—remains poorly understood and inconsistently documented, especially when using objective clinical and instrumental measures.

This study aims to examine simple snoring rather than OSA, as a potentially modifiable factor influencing masticatory muscle pain severity in patients with myogenous TMD. By specifically examining patients without clinically confirmed OSA, the research seeks to determine whether snoring alone, independent of more severe forms of SDB, is associated with increased masticatory muscle pain. This distinction is crucial for the early detection and treatment of TMD patients who might otherwise be overlooked in sleep-related evaluations.

## 2. Materials and methods

The details regarding the study design are presented in Fig. 1.

**Fig. 1.** Flow diagram illustrating the study process, including participant examination and data collection. OSA: obstructive sleep apnea; TMD: temporomandibular disorders; NRS: numeric rating scale; EEG: electroencephalograms; EOG: electrooculogram; EMG: electromyography; ECG: electrocardiogram; REM: rapid eye movement; AHI: apnea-hypopnea index; DC: diagnostic criteria.

### 2.1 Study design

This cross-sectional prospective study was conducted in the Sleep Laboratory of the Department of Internal Medicine, Occupational Diseases, Hypertension, and Clinical Oncology at Wroclaw Medical University (Wroclaw, Poland), as well as in the Department of Experimental Dentistry at Wroclaw Medical University. The study included patients who were admitted to the Sleep Laboratory for polysomnographic examination from the Outpatient Clinic for Temporomandibular Disorders operating within the Department of Experimental Dentistry at Wroclaw Medical University, due to reported snoring and a diagnosis of probable bruxism (Fig. 1). All participants provided informed consent. The study was approved by the Ethics Committee of Wroclaw Medical University (ID: KB-195/2017, KB-794/2019) and conducted in accordance with the Declaration of Helsinki. Clinical trial registration information is available at www.ClinicalTrials.gov (identifiers: NCT03083405, NCT04214561). The study was reported in accordance with the Strengthening the Reporting of Observational Studies in Epidemiology (STROBE) Statement for cross-sectional studies [30]. The current analysis represents an additional evaluation of a dataset originally collected prospectively for a separate, previously registered study.

### 2.2 Participants

The inclusion criteria were as follows: willingness to participate; age ≥18 years; presence of myogenous TMD (including local myalgia, myofascial pain, and myofascial pain with referral) in accordance with the Diagnostic Criteria for Temporomandibular Disorders (DC/TMD) Axis I [31]; probable bruxism defined according to the International Consensus on the Assessment of Bruxism [32]; and reported snoring [33].

The exclusion criteria included the following: genetic disorders; inability to undergo polysomnography (PSG); apnea-hypopnea index (AHI) >5; diagnosed OSA; secondary bruxism associated with neurological conditions; neurological disorders and/or neuropathic pain (including primary headaches assessed using the Third Edition of the International Classification of Headache Disorders) [34]; coexistence of respiratory insufficiency and active inflammation; active malignancy; severe mental disorders or significant mental (including genetic) disabilities; TMD types other than myogenous TMD; use of an occlusal splint or any muscle relaxation device within 2 weeks before PSG; alcohol addiction; pregnancy or confinement; treatment with or addiction to any analgesic agents and/or drugs affecting the function of the nervous system, muscles, or breathing; and, importantly, refusal to provide consent for participation.

### 2.3 Data sources/measurement

Participants were recruited from patients attending the Outpatient Clinic for Temporomandibular Disorders at the Department of Experimental Dentistry, Wroclaw Medical University, between 2017 and 2022. Each patient underwent a comprehensive medical interview and dental examination, with particular attention to self-reported simple snoring, including reports from bed partners. Additionally, the temporomandibular joints and masticatory muscles were assessed according to the DC/TMD Axis I [31]. Evaluation for sleep bruxism or awake bruxism was performed using multiple approaches, including patient questionnaires and structured interviews. The examination included the identification of clinical indicators such as alterations in dental hard tissues and oral mucosa, tooth wear, tongue scalloping, and the presence of a linea alba. All assessments were conducted by a dentist trained in DC/TMD with a minimum of five years of experience in TMD evaluation and management.

Patients reporting simple snoring, as defined by the Third Edition of the International Classification of Sleep Disorders by the American Academy of Sleep Medicine [33], were referred to the Sleep Laboratory at the Department and Clinic of Internal Medicine, Occupational Diseases, Hypertension, and Clinical Oncology, Wroclaw Medical University, where they underwent a single-night video-polysomnography (vPSG) to confirm the presence of simple snoring.

#### 2.3.1 Masticatory muscles pain intensity assessment

Masticatory muscle pain was assessed individually for each patient within 30 days before PSG through palpation performed by an experienced dentist. The right and left temporal muscles, as well as the right and left masseter muscles, were examined separately in accordance with the DC/TMD protocol, with each patient evaluated only once. This separate assessment of muscles on each side followed DC/TMD guidelines.

During the examination, participants reported their pain intensity using the Numeric Rating Scale (NRS), a 0–10 scale widely used in pain assessment, where 0 represents “no pain” and 10 corresponds to “unbearable pain”. NRS scores of ≤3 were classified as mild pain, 4–6 as moderate pain, and ≥7 as severe pain [35]. For each muscle, a global NRS score was calculated as the average of the nine palpation points. For statistical analyses, additional categorical classifications were applied: “no pain”, “mild pain at most” (combining no pain and mild pain), and “severe pain”.

#### 2.3.2 Polysomnographic assessment

All patients underwent an overnight polysomnographic evaluation using the Nox-A1 device (Nox Medical, Reykjavik, Iceland) at the Sleep Laboratory of the Department of Internal Medicine, Occupational Diseases, Hypertension, and Clinical Oncology, Wroclaw Medical University (Wroclaw, Poland). Electroencephalograms (EEGs) were recorded according to the American Academy of Sleep Medicine (AASM)-recommended montages. Eight EEG and electrooculogram (EOG) channels were used (F4-M1, C4-M1, O2-M1, F3-M2, C3-M2, O1-M2, E1-M2, E2-M2), with electrode placement following the International 10–20 System [36]. Three electrodes were positioned to record chin electromyography (EMG) at the mental and submental sites. Respiratory effort was monitored using respiratory inductance plethysmography (RIP) belts placed around the thorax and abdomen. Cardiac activity was recorded using a modified electrocardiogram (ECG) Lead II. Hypopneas were detected using a nasal pressure transducer, and apneas were identified using an oronasal thermal airflow sensor.

Polysomnograms were scored in 30-s epochs, initially by automated analysis and subsequently reviewed manually by a certified polysomnographer. Sleep stages were classified according to the 2013 AASM Task Force criteria [33]. PSG outcomes included sleep latency (SL), REM latency, total sleep time, sleep efficiency, and the proportions of N1, N2, N3, and REM sleep. Sleep position was determined automatically using integrated position sensors.

#### 2.3.3 Snoring examination

Snoring was monitored using an acoustic sensor and a nasal pressure transducer. Sounds were classified as snore events if they were synchronized with respiration and exhibited a distinct audible oscillatory component. The full audio signal was recorded using Noxturnal software (version 5.1.3, Nox Medical, Reykjavik, Iceland) at a sampling rate of 8000 Hz, then downsampled to 100 Hz and processed with decibels relative to carrier (dBc) weighting to generate an audio envelope in decibels (dB). Consecutive individual snores were grouped and scored as snore trains, all of which were automatically recorded [34]. Both inspiratory and expiratory snores were included in the analysis. Nonsnore sounds—such as coughing, groaning, bruxism-related noises, duvet noise, movement artifacts, and large breathing sounds without vibration were excluded.

### 2.4 Statistical analysis

Group size was determined using a sample size calculator. The required sample size was calculated based on a population size of 3,000,000, an assumed population proportion of 12%, a 95% confidence level (*Z* = 1.96), and a maximum allowable margin of error of 5%. Using a standard formula for sample size estimation in finite populations, the minimum number of study participants needed to ensure statistically valid results was determined to be 163. To align with the association-based objective of the study, we also calculated the detectable effect size for correlation analyses in the achieved sample. With the realized sample (n = 184) and a two-sided α = 0.05, the study had 80% power to detect Pearson correlations of *r* ≥ 0.205 (Fisher’s z method). Under comparable conditions, the power for Spearman’s rank correlations is expected to be similar.

Data were divided according to four snoring-related parameters: Snore Index, Snore Train Percentage, Number of Snore Trains per Hour, and Average Audio Volume (dBc). For each variable, the dataset was split into two subgroups based on the median and into four subgroups according to quartile distribution. Variables are presented as mean ± standard deviation (SD). Normality was assessed using the Shapiro-Wilk test, and equality of variances was evaluated with Levene’s test. Comparisons between two groups were performed using Student’s *t*-test. When assumptions for parametric testing were not met, the Mann-Whitney U test was applied. For comparisons involving four groups, a one-way analysis of variance (ANOVA) was conducted; if the assumption of equal variances was violated, Welch’s ANOVA was applied. When overall significance was observed, appropriate *post hoc* tests (Tukey honestly significant difference (HSD) or Games-Howell) were performed to determine group differences. Correlations between continuous variables were evaluated using the Pearson correlation coefficient, while Spearman’s rank correlation was applied to qualitative or non-normally distributed data.

As a supplementary analysis, a multivariable linear regression was performed with pain intensity as the dependent variable to explore the influence of potential confounders, including bruxism (BEI), body mass index (BMI), Snore Index, Snore Train Percentage, Number of Snore Trains per Hour, and Average Audio Volume (dBc). Statistical analyses were conducted using Statistica software, version 13.3 (StatSoft, Krakow, Poland), and Jamovi (version 2.4.19, The Jamovi Project, Sydney, Australia). A *p*-value < 0.05 was considered statistically significant. Directional inference applies only to the supplementary multivariable regression, which specified pain as the outcome and therefore evaluated the direction from snoring to pain; the reverse direction was not modeled. Correlation and subgroup analyses remain direction-agnostic.

## 3. Results

### 3.1 Study population

The study included 184 participants with an average age of 33.92 ± 10.05 years. Females comprised 71.2% (n = 131) of the group. The average BMI was 24.00 ± 3.84 kg/m^2^. Comorbidities were rare, with hypertension present in 5.4% (n = 10) of participants. None had a history of diabetes, ischemic heart disease, myocardial infarction, or stroke (Table 1).

**Table 1.** Demographic and clinical characteristics of the study population (n = 184).

Parameter		Value
Demographics		
	Age (yr), mean ± SD	33.92 ± 10.05
	Female gender, n (%)	131 (71.2%)
	Male gender, n (%)	53 (28.8%)
	BMI (kg/m^2^), mean ± SD	24.00 ± 3.84
Comorbidities		
	Hypertension, n (%)	10 (5.4%)
	Diabetes mellitus, n (%)	0 (0%)
	Ischemic heart disease, n (%)	0 (0%)
	Myocardial infarction, n (%)	0 (0%)
	Stroke, n (%)	0 (0%)

*SD: standard deviation; BMI: body mass index.*

### 3.2 Correlation analysis

Overall, the study demonstrated that as muscle pain increased, snore parameter values tended to decrease.

#### 3.2.1 Main snoring parameters and muscle pain intensity

Correlation analysis revealed no statistically significant associations between the main snoring parameters (snore index, snore train percentage, number of snore trains per hour, and average audio volume) and masticatory muscle pain intensity across all four muscle locations studied (*p * > 0.05 for all comparisons) (Table 2).

**Table 2.** Correlations between main snoring parameters and masticatory muscle pain intensity (n = 184).

Snoring parameter		Right masseter	Left masseter	Right temporalis	Left temporalis
Snore index					
	Correlation coefficient (*r*)	−0.157	−0.110	−0.148	−0.102
	*p*-value	0.056	0.184	0.072	0.220
Snore train percentage					
	Correlation coefficient (*r*)	−0.160	−0.103	−0.157	−0.109
	*p*-value	0.052	0.213	0.057	0.188
Number of snore trains per hour					
	Correlation coefficient (*r*)	−0.099	−0.058	−0.097	−0.044
	*p*-value	0.233	0.485	0.241	0.593
Average audio volume (dBc)					
	Correlation coefficient (*r*)	−0.060	−0.014	−0.102	−0.029
	*p*-value	0.466	0.866	0.217	0.727

*dBc: decibels relative to carrier.*

#### 3.2.2 Other snoring parameters and pain intensity in studied locations

Among all the snoring parameters analyzed, 18 correlations reached statistical significance (*p * < 0.05), involving 14 distinct parameters (Table 3). Notably, all significant correlations were negative, except one positive correlation between minimum audio volume in the nonsupine position and left masseter pain (*r* = 0.164, *p* = 0.046) (Table 3).

**Table 3.** Statistically significant correlations between snoring parameters and pain (n = 184).

Snoring parameter		Location	Correlation coefficient (*r*)	*p*-value
Nonsupine position parameters				
	Snore index (nonsupine)	Left masseter	−0.227	0.005**
	Snore index in N2 (nonsupine)	Left masseter	−0.180	0.029*
	Snore index in REM (nonsupine)	Left masseter	−0.211	0.010*
	Snore train percentage (nonsupine)	Left masseter	−0.176	0.033*
	Snore train percentage in N2 (nonsupine)	Left masseter	−0.168	0.042*
	Snore train percentage in N3 (nonsupine)	Right temporalis	−0.164	0.047*
	Snore train percentage in REM (nonsupine)	Left masseter	−0.168	0.041*
		Right temporalis	−0.181	0.028*
		Left temporalis	−0.210	0.010*
	Percent of snores >80 dBc (nonsupine)	Right temporalis	−0.173	0.035*
		Left temporalis	−0.181	0.028*
	Minimum audio volume (nonsupine)^†^	Left masseter	0.164	0.046*
Supine position parameters				
	Average audio volume (supine)	Right masseter	−0.199	0.015*
	Maximum audio volume (supine)	Right masseter	−0.199	0.015*
	Minimum audio volume (supine)	Right masseter	−0.168	0.041*
Total (all positions) parameters				
	Snore train percentage in N3 (total)	Right masseter	−0.162	0.049*
	Percent of snores >80 dBc (total)	Left masseter	−0.176	0.032*
		Left temporalis	−0.171	0.038*

*p < 0.05; **p < 0.01; ^†^Only positive correlation found. dBc: decibels relative to carrier; N3: Nonrapid eye movement 3; N2: Nonrapid eye movement 2; REM: Rapid eye movement.

##### 3.2.2.1 Body position-specific findings

Most of the significant correlations (11 out of 18) involved snoring parameters measured in the nonsupine position. The strongest correlation was observed between the snore index in the nonsupine position and left masseter pain (*r* = −0.227, *p* = 0.005), which was the only correlation with *p * < 0.01. Nonsupine snoring parameters were associated primarily with left-sided muscle pain, accounting for 6 of the 11 significant nonsupine correlations.

##### 3.2.2.2 Sleep stage-specific associations

Snoring during REM sleep in the nonsupine position showed the most widespread associations, correlating significantly with pain at three of the four measured sites (left masseter: *r* = −0.168, *p* = 0.041; right temporalis: *r* = −0.181, *p* = 0.028; left temporalis: *r* = −0.210, *p* = 0.010). Additionally, snoring intensity during N3 sleep demonstrated selective associations with right-sided pain (Table 3).

##### 3.2.2.3 Acoustic parameters

Supine-position snoring loudness parameters (average, maximum, and minimum audio volume) showed exclusive associations with right masseter pain (*r* = −0.168 to −0.199, *p* = 0.015–0.041). The percentage of loud snores (>80 dBc) correlated significantly with bilateral temporal muscle pain, but not with masseter pain (Table 3).

##### 3.2.2.4 Laterality patterns

The left masseter muscle exhibited the highest number of significant correlations (n = 8), followed by the right temporalis (n = 3), the left temporalis (n = 3), and the right masseter (n = 4). This left-sided dominance was particularly evident in correlations with nonsupine snoring parameters (Table 3).

#### 3.2.3 Analyses based on bilaterality of pain

Table 4 presents statistically significant negative correlations between bilateral masseter pain, bilateral temporalis pain, and selected snoring parameters.

**Table 4.** Statistically significant correlations between snoring parameters and the bilaterality of pain.

Comparisons	Spearman’s rang correlation		
	N	*r*	*p*-value
Bilateral masseter and percent of snores >70 dBc (Supine)	184	−0.168	0.0230*
Bilateral masseter and percent of snores >70 dBc (Total)	184	−0.167	0.0230*
Bilateral temporalis and snore index in REM (nonsupine)	177	−0.149	0.0481*
Bilateral masseter and snore index in N2 (supine)	183	−0.146	0.0480*

*p < 0.05; N2: Nonrapid eye movement 2; REM: Rapid eye movement; dBc: decibels relative to carrier.

### 3.3 Group comparisons

Participants were grouped based on median and quartile divisions according to four main snoring parameters: snore index, snore train percentage, number of snore trains per hour, and average audio volume.

#### 3.3.1 Median-based analysis

When comparing groups based on the snore index (median cutoff ≤25.3 *vs.* >25.3), individuals with a higher snore index showed significantly lower pain intensity in the right masseter muscle compared to those with a lower snore index (*p* = 0.047). Additionally, bilateral masseter pain was significantly lower in the high snore index group compared to the low group (*p* = 0.014). No significant differences were observed for the left masseter, the temporalis muscles, or bilateral temporalis pain (*p * > 0.05 for all comparisons).

In the analysis stratified by snore train percentage (median ≤0.95 *vs.* >0.95), individuals with a higher snore train percentage reported significantly lower NRS scores for the right masseter muscle compared to those with lower percentages (*p* = 0.025). Pain in the right temporalis muscle was also significantly lower in the high snore train percentage group (*p* = 0.032). No significant differences were found for the left masseter, bilateral masseter, left temporalis, or bilateral temporalis (*p * > 0.05 for all comparisons).

When grouped by the number of snore trains per hour (median ≤1.20 *vs.* >1.20), participants with a higher number of snore trains exhibited lower pain intensity in the right masseter compared to the low group (*p* = 0.050). No significant differences were observed for the remaining muscle groups or bilaterality parameters (*p * > 0.05 for all comparisons).

Finally, no statistically significant differences were detected between groups divided by average audio volume (median ≤65.6 *vs.* >65.6) for any of the assessed masticatory muscle pain variables (*p * > 0.05 for all comparisons). Median-based analysis results are presented in Table 5.

**Table 5.** Comparison of masticatory muscle pain intensity and bilaterality parameters between groups, median-stratified by snoring parameters (snore index, snore train percentage, number of snore trains per hour, and average audio volume).

Variable	n	Average	SD	n	Average	SD	*p*-value
	Low snore index (median ≤25.3)			High snore index (median >25.3)			
Right masseter	92	5.163	2.715	92	4.272	3.292	0.047
Left masseter		4.989	2.633		4.293	3.147	ns
Bilateral masseter		0.913	0.283		0.783	0.415	0.014
Right temporalis		5.500	2.337		4.804	3.103	ns
Left temporalis		5.250	2.197		5.022	2.821	ns
Bilateral temporalis		0.880	0.326		0.848	0.361	ns
	Low snore train percentage (median ≤0.95)			High snore train percentage (median >0.95)			
Right masseter	92	5.217	2.745	92	4.217	3.251	0.025
Left masseter		4.978	2.660		4.304	3.126	ns
Bilateral masseter		0.891	0.313		0.804	0.399	ns
Right temporalis		5.587	2.391		4.717	3.039	0.032
Left temporalis		5.261	2.208		5.011	2.811	ns
Bilateral temporalis		0.859	0.350		0.870	0.339	ns
	Low number of snore trains per hour (median ≤1.20)			High number of snore trains per hour (median >1.20)			
Right masseter	94	5.170	2.838	89	4.292	3.174	ns
Left masseter		4.798	2.804		4.528	3.012	ns
Bilateral masseter		0.883	0.323		0.809	0.395	ns
Right temporalis		5.468	2.500		4.876	2.961	ns
Left temporalis		5.234	2.269		5.090	2.737	ns
Bilateral temporalis		0.840	0.368		0.888	0.318	ns
	Low average audio volume (median ≤65.6)			High average audio volume (median >65.6)			
Right masseter	92	4.880	2.889	87	4.460	3.241	ns
Left masseter		4.783	2.812		4.402	3.059	ns
Bilateral masseter		0.870	0.339		0.816	0.390	ns
Right temporalis		5.152	2.813		5.092	2.769	ns
Left temporalis		5.076	2.437		5.138	2.660	ns
Bilateral temporalis		0.848	0.361		0.874	0.334	ns

*SD: standard deviation; ns: non-significant.*

#### 3.3.2 Quartile-based analysis

When analyzing groups based on snore index quartiles (mild: 8.39–25.30; moderate: 25.31–87.25; severe: >87.25), participants with a severe snore index showed significantly lower pain intensity in the right masseter muscle compared to those with a mild snore index (*p* = 0.012). Bilateral masseter pain was significantly lower in both the moderate and severe snore index groups compared with the mild group (*p* = 0.042 for both). Pain in the right temporalis muscle was also significantly lower in the severe snore index group relative to the mild group (*p* = 0.038). No significant differences were observed for the left masseter, left temporalis, or bilateral temporalis across snore index quartiles (*p * > 0.05 for all).

In the analysis stratified by snore train percentage quartiles (Q1 ≤0.10; mild: 0.11–0.95; moderate: 0.96–7.30; severe: >7.30), participants with severe snore train percentages reported significantly lower NRS scores for the right masseter muscle compared to those in the mild group (*p* = 0.021). No significant differences were found for the left masseter, bilateral masseter, left temporalis, or bilateral temporalis across snore train percentage quartiles (*p * > 0.05 for all).

Finally, in the analysis of average audio volume quartiles (very low: ≤64.2; low: 64.3–65.6; moderate: 65.7–67.9; high: >67.9), participants with high audio volumes showed significantly lower pain intensity in the right masseter muscle compared to those with moderate volumes (*p* = 0.044). Likewise, right temporalis muscle pain was significantly lower in the high audio volume group compared to the moderate group (*p* = 0.039). No statistically significant differences were detected for the left masseter, bilateral masseter, left temporalis, or bilateral temporalis across audio volume quartiles (*p * > 0.05 for all). Data for the quartile-based analyses are presented in Tables 6 and 7.

**Table 6.** Comparison of masticatory muscle pain intensity and bilaterality parameters between groups, median-stratified by snoring parameters (snore index, snore train percentage, number of snore trains per hour, and average audio volume).

Variable	n	Average	SD	n	Average	SD	n	Average	SD
	Mild snore index (8.39–25.30)			Moderate snore index (25.31–87.25)			Severe snore index (>87.25)		
Right masseter	46	5.478	2.483	46	4.652	3.315	46	3.891	3.260
Left masseter		5.326	2.395		4.391	3.228		4.195	3.095
Bilateral masseter		0.935	0.250		0.782	0.417		0.782	0.417
Right temporalis		5.565	2.491		5.239	3.226		4.369	2.946
Left temporalis		5.348	2.321		5.152	2.951		4.891	2.709
Bilateral temporalis		0.913	0.285		0.891	0.315		0.804	0.401
	Mild snore train percentage (0.11–0.95)			Moderate snore train percentage (0.96–7.30)			Severe snore train percentage (>7.3)		
Right masseter	34	5.059	2.806	47	4.510	3.256	45	3.911	3.253
Left masseter		4.824	2.779		4.255	3.172		4.355	3.112
Bilateral masseter		0.882	0.327		0.787	0.413		0.822	0.386
Right temporalis		5.441	2.743		4.936	3.088		4.488	3.004
Left temporalis		5.059	2.510		5.127	2.901		4.888	2.740
Bilateral temporalis		0.882	0.327		0.893	0.311		0.844	0.366
	Mild number of snore trains per hour (0.31–1.20)			Moderate number of snore trains per hour (1.21–5.7)			Severe number of snore trains per hour (>5.7)		
Right masseter	43	4.977	2.948	43	4.720	3.194	46	3.891	3.135
Left masseter		4.488	3.003		4.907	2.982		4.173	3.028
Bilateral masseter		0.884	0.324		0.814	0.393		0.804	0.401
Right temporalis		5.256	2.761		5.255	2.829		4.521	3.067
Left temporalis		5.140	2.484		5.302	2.773		4.891	2.718
Bilateral temporalis		0.860	0.351		0.953	0.213		0.826	0.383
	Low average audio volume (64.3–65.6)			Moderate average audio volume (65.7–67.9)			High average audio volume (>67.9)		
Right masseter	49	5.000	2.972	44	5.114	3.036	43	3.790	3.342
Left masseter		5.082	2.753		4.886	3.021		3.907	3.053
Bilateral masseter		0.878	0.331		0.841	0.370		0.790	0.411
Right temporalis		5.204	2.944		5.705	2.378		4.465	3.018
Left temporalis		5.143	2.541		5.568	2.528		4.697	2.747
Bilateral temporalis		0.816	0.391		0.932	0.255		0.814	0.393

*SD: standard deviation.*

**Table 7.** Statistically significant quartile-based comparisons.

Variable	Comparison	*p*-value
Right masseter		
	Mild *vs.* severe snore index	0.012*
	Mild *vs.* severe snore train percentage	0.021*
	Moderate *vs.* high average audio volume	0.044*
Bilateral masseter		
	Mild *vs.* moderate snore index	0.042*
	Mild *vs.* severe snore index	0.042*
Right temporalis		
	Mild *vs.* severe snore index	0.038*
	Moderate *vs.* high average audio volume	0.039*

*p < 0.05.

### 3.4 Multivariate analysis

Multiple linear regression analysis was conducted to assess the independent relationships between snoring parameters, sleep bruxism, and BMI with right masseter pain while accounting for potential confounders (Table 8). The overall model for right masseter pain intensity—selected as the outcome due to its strongest univariate correlations—showed no independent associations with bruxism, BMI, snore index, snore train percentage, number of snore trains per hour, or average audio volume (*p * > 0.05 for all comparisons) (Table 8).

**Table 8.** Multiple linear regression analysis for right masseter pain (n = 123).

Independent variable	*p*-value	Clinical significance
BMI	0.395	ns
Snore index	0.172	ns
Snore train percentage	0.127	ns
Number of snore trains per hour	0.903	ns
Average audio volume (dBc)	0.386	ns

*dBc: decibels relative to carrier; BMI: Body mass index; ns: not significant.*

## 4. Discussion

From a clinical perspective, this study has important implications: it may support the inclusion of simple snoring in routine TMD screening protocols and promote interdisciplinary management strategies involving dental, sleep, and pain specialists. From a scientific perspective, the study addresses an underexplored area, offering new insights into the relationship between upper airway resistance, muscle activity, and chronic pain. Ultimately, these findings may enhance understanding of TMD etiology and contribute to the development of more precise, multifactorial treatment approaches.

The relationship between sleep-related breathing disturbances and TMD continues to be investigated. Our study provides new evidence regarding the association between snoring and masticatory muscle pain. The evaluation demonstrated an absence of statistically significant associations between the primary snoring parameters and pain levels across the examined masticatory muscles. In light of this, the discussion focuses on other findings that may provide a deeper understanding of the interplay between sleep-related variables and TMD.

Previous studies report that sleep-disordered breathing, including OSA, may increase the risk of developing TMD through mechanisms such as sleep fragmentation, increased muscle tone, and systemic inflammation [16, 37, 38, 39, 40, 41, 42, 43]. Moreover, SB has been shown to modify sleep architecture, with significant differences found between bruxers and non-bruxers; sleep bruxism was also more frequent in individuals reporting alcohol intake than in those who abstained [44]. Huang *et al*. [45] demonstrated that increased age, male gender, daily alcohol consumption, depression, daytime sleepiness, and high gastroesophageal reflux disease (GERD) risk are strongly associated with increased OSA risk in patients with SB, whereas high TMD-pain risk and chronic pain are strongly linked to decreased OSA risk in the same population. These findings suggest that both sleep bruxism and breathing disorders are associated with numerous life and behavioral factors, which—when genetically determined—may be difficult to modify.

Similar conclusions were reported by Lee *et al*. [46], who emphasized the importance of considering sex-based differences in the evaluation of sleep-related disorders, including snoring and OSA, among patients with TMD. The authors highlighted the potential clinical utility of Mallampati scoring as a preliminary screening tool before PSG, which may assist clinicians in identifying patients at risk of snoring and OSA. The study further underscored the importance of integrating sleep-related and biopsychosocial factors in the comprehensive diagnosis and management of TMD [46]. The study by Emodi-Perlman *et al*. [47] highlights notable sex-related differences in the effectiveness of screening tools for assessing the risk of OSA. Fatigue scores measured using the Fatigue Assessment Scale (FAS) were significantly predictive of moderate to severe OSA in males but not in females. Although females reported higher average fatigue levels, fatigue was significantly associated with OSA only in males. This suggests that self-reported fatigue symptoms in males may reflect physiological indicators of sleep-disordered breathing, whereas in females, fatigue may stem from multifactorial origins unrelated to OSA severity [47]. Another important finding, reported by Smardz *et al*. [48], showed that tonic muscle contractions may both contribute to and result from respiratory events. This suggests that bruxism may function either as a defensive physiological response or an initiating factor to an episode of apnea.

The relationship between OSA and SB appears to be influenced by the severity of OSA. As demonstrated by Martynowicz* et al*. [49], mild to moderate OSA is associated with the presence of SB in patients at increased risk for OSA. Focusing specifically on primary snoring, independent of obstructive sleep apnea, the study by Michalek-Zrabkowska *et al.* [26] demonstrated a positive correlation between snore intensity and phasic bruxism in both supine and nonsupine sleep positions. Body position was also shown to influence the intensity of both snoring and bruxism. These findings provide evidence supporting a relationship between hypoxia and SB in individuals with simple snoring [26]. Numerous factors influence the development and modulation of TMD, particularly its association with snoring and OSA. As mentioned previously, these may result from environmental influences, patient behaviors, and habitual patterns. However, the genetic component remains an open question, which could provide an alternative treatment pathway. According to the most recent data presented by Wu *et al*. [50], it has been demonstrated that there is limited but novel genetic evidence supporting a potential causal link between snoring and a decreased risk of developing TMD. The further development of this line of research may contribute to refining and optimizing therapeutic approaches. Conversely, it does not substantiate an effect of TMD on the likelihood of snoring [50]. Consistent with these findings, our study revealed limited evidence supporting an association between snoring and TMD-related symptoms.

In the present study, no statistically significant correlations were found between the primary snoring parameters and the intensity of masticatory muscle pain across the evaluated masticatory muscle groups. Interestingly, several specific snoring characteristics, particularly those recorded during REM sleep and in nonsupine positions, showed statistically significant negative correlations with pain. These findings suggest that snoring, under certain sleep conditions, may exert a modulatory or potentially protective effect on muscle-related TMD symptoms.

This protective association appeared to be lateralized, with more consistent associations observed in the right masticatory muscles, particularly the right masseter. Supine snoring variables were more closely related to right-sided symptoms, whereas nonsupine parameters were linked to pain on the left. This laterality may reflect the influence of habitual sleep posture, potentially leading to asymmetric neuromuscular loading. Prior research has emphasized the biomechanical relevance of body position in sleep-disordered breathing, such as obstructive sleep apnea [51]. Additionally, findings from Minervini *et al.* [52] further identified a correlation between body posture and TMD, supporting the hypothesis that sleep positioning may influence masticatory muscle pain. Future studies using high-resolution postural tracking may help clarify this relationship further.

Notably, the literature on this subject remains inconsistent. Some studies suggest that snoring and OSA act as risk factors for TMD [53], while others report neutral or even protective associations [49, 54]. Methodological limitations may contribute to these discrepancies. Many studies rely on self-reported snoring or fail to differentiate between primary snoring and OSA. This distinction is crucial, as untreated OSA can affect sleep quality, promote systemic inflammation, and alter pain thresholds—all of which may confound study outcomes [55, 56]. Additionally, only a limited number of studies have employed PSG, the gold standard for assessing sleep architecture and breathing disturbances. The lack of objective diagnostic methods and inadequate control for OSA may have introduce misclassification bias, contributing to inconsistent findings.

Several limitations of this study should be acknowledged. First, the polysomnographic recordings were conducted without an adaptation night, which may have introduced a first-night effect and influenced sleep architecture, potentially affecting the accuracy of sleep-related measurements. Another potential limitation is the exclusion of patients taking analgesics, which may have influenced the assessment of pain intensity and distribution and could have resulted in underestimation of true pain levels. Additionally, the cross-sectional design limits the ability to establish causality, as all data were collected at a single time point. There was also no assessment of pain duration, frequency, or the number of sites with myogenous pain. Previous studies have reported sex differences in snoring intensity; therefore, the absence of gender-stratified analysis represents another important limitation. Finally, the predominance of young, healthy female participants may restrict the generalizability of our findings. Given that age and male gender are important risk factors for snoring and OSA, the lack of inclusion of these variables limits broader applicability.

An additional limitation concerns the generalizability of the results, as many of the observed correlation coefficients (*r*-values) did not reach the threshold of 0.205, which was determined during the sample size calculation. Consequently, these findings should be interpreted with caution and may not be fully generalizable to the broader population.

This study has several methodological strengths that enhance the reliability and validity of its results. First, no occlusal splints or muscle relaxation devices were used before or during the polysomnographic recordings, eliminating potential confounding effects on masticatory muscle activity during sleep. Second, the study included a relatively large sample size, which improves statistical power and supports the generalizability of the findings. A key strength is the objective assessment of snoring, based on overnight polysomnography rather than subjective self-reports or questionnaires, as is common in previous research. This approach provides a more accurate and reproducible measurement of snoring. Additionally, to isolate the effects of primary snoring, participants with clinically significant obstructive sleep apnea (AHI ≥5) were excluded, thereby reducing potential confounding from apneic events. Finally, all procedures, including PSG scoring, clinical evaluations, and diagnostic criteria—were conducted using standardized and validated protocols, ensuring methodological consistency and rigor throughout the study.

The exact mechanism underlying the observed decrease in right-sided masticatory muscle pain with increasing snoring intensity remains unknown. This study was unable to determine the cause of this relationship, and current literature has not provided a conclusive explanation. Further research is needed to investigate this phenomenon and elucidate the potential biological mechanisms involved.

## 5. Conclusions

The findings of this study indicate a complex and nuanced relationship between snoring and myogenous TMD. Individuals who snore and have myogenous TMD tended to experience lower masticatory muscle pain compared with non-snorers with the condition. However, snoring in this group was associated with a more asymmetrical distribution of muscle pain. Additionally, the severity of TMD-related muscle pain was linked to the intensity of snoring rather than its loudness, suggesting a possible physiological connection.

Overall, the association between snoring and myogenous TMD appears weak; however, the potential protective or modulatory influence of snoring on masticatory muscle pain cannot be dismissed. These findings underscore the need for further investigation through prospective, longitudinal studies to clarify the underlying mechanisms and causal pathways.
